# Stabilization of LKB1 and Akt by neddylation regulates energy metabolism in liver cancer

**DOI:** 10.18632/oncotarget.3191

**Published:** 2014-12-11

**Authors:** Lucía Barbier-Torres, Teresa C. Delgado, Juan L. García-Rodríguez, Imanol Zubiete-Franco, David Fernández-Ramos, Xabier Buqué, Ainara Cano, Virginia Gutiérrez-de Juan, Itziar Fernández-Domínguez, Fernando Lopitz-Otsoa, Pablo Fernández-Tussy, Loreto Boix, Jordi Bruix, Erica Villa, Azucena Castro, Shelly C. Lu, Patricia Aspichueta, Dimitris Xirodimas, Marta Varela-Rey, José M. Mato, Naiara Beraza, María L. Martínez-Chantar

**Affiliations:** ^1^ CIC bioGUNE, Centro de Investigación Biomédica en Red de Enfermedades Hepáticas y Digestivas (CIBERehd), Bizkaia Science and Technology Park, Derio, Bizkaia, Spain; ^2^ Department of Physiology, University of the Basque Country UPV/EHU, Faculty of Medicine and Dentistry, Leioa, Bizkaia, Spain; ^3^ OWL, Bizkaia Science and Technology Park, Derio, Bizkaia, Spain; ^4^ Barcelona-Clínic Liver Cancer Group, Institut d’Investigacions Biomèdiques August Pi I Sunyer, Liver Unit, Hospital Clínic, Universitat de Barcelona, Barcelona, Catalonia, Spain; ^5^ Centro de Investigación Biomédica en Red de Enfermedades Hepáticas y Digestivas (CIBERehd), Instituto de Salud Carlos III, Madrid, Spain; ^6^ Department of Gastroenterology, Azienda Ospedaliero-Universitaria & University of Modena and Reggio Emilia, Modena, Italy; ^7^ Division of Gastroenterology, Cedars-Sinai Medical Center, Los Angeles, CA, USC Research Center for Liver Diseases, Keck School of Medicine, Los Angeles, CA, USA; ^8^ Centre de Recherche de Biochimie Macromoléculaire, Montpellier, France

**Keywords:** Neddylation, Cancer metabolism, Hepatocellular Carcinoma, LKB1, Akt

## Abstract

The current view of cancer progression highlights that cancer cells must undergo through a post-translational regulation and metabolic reprogramming to progress in an unfriendly environment. In here, the importance of neddylation modification in liver cancer was investigated. We found that hepatic neddylation was specifically enriched in liver cancer patients with bad prognosis. In addition, the treatment with the neddylation inhibitor MLN4924 in *Phb1*-KO mice, an animal model of hepatocellular carcinoma showing elevated neddylation, reverted the malignant phenotype. Tumor cell death *in vivo* translating into liver tumor regression was associated with augmented phosphatidylcholine synthesis by the PEMT pathway, known as a liver-specific tumor suppressor, and restored mitochondrial function and TCA cycle flux. Otherwise, in protumoral hepatocytes, neddylation inhibition resulted in metabolic reprogramming rendering a decrease in oxidative phosphorylation and concomitant tumor cell apoptosis. Moreover, Akt and LKB1, hallmarks of proliferative metabolism, were altered in liver cancer being new targets of neddylation. Importantly, we show that neddylation-induced metabolic reprogramming and apoptosis were dependent on LKB1 and Akt stabilization. Overall, our results implicate neddylation/signaling/metabolism, partly mediated by LKB1 and Akt, in the development of liver cancer, paving the way for novel therapeutic approaches targeting neddylation in hepatocellular carcinoma.

## INTRODUCTION

Hepatocellular carcinoma (HCC) is the fifth most common cancer worldwide and the third leading cause of death [1]. HCC etiology is very complex with multiple and distinct signaling pathways converging in malignant transformation [2]. The current view of cancer progression supports that cancer cells must undergo through a post-translational modification (PTM) regulation and a metabolic switch or reprogramming in order to progress in an unfriendly environment [3]. Neddylation is a PTM by which the ubiquitin-like protein, neural precursor cell expressed, developmentally downregulated 8 (Nedd8) is conjugated to its target proteins. Since Nedd8 is a key regulator of cell growth, viability and malignant transformation [4], neddylation inhibition could prove to be an effective anti-cancer therapy. Although the cullin-RING family of E3 ubiquitin-ligases are the best-characterized Nedd8 substrates, other proteins have been reported to be modified by Nedd8, including p53, murine double minute 2 (Mdm2), epidermal growth factor receptor, VHL tumour suppressor protein, L11, and other RNA binding proteins such as human antigen R (HuR), a hallmark of liver tumor progression [5-8]. Although not described to date, several other proteins could be potential neddylation targets.

Liver kinase B1 (LKB1) and Akt kinases are proteins known to play an important role in metabolism regulation [9]. LKB1 directly phosphorylates and activates AMP-activated protein kinase (AMPK), a central metabolic sensor [10]. Previously, we have shown that LKB1 activity, highly representative in HCC patients with poor outcome, has a surprising pro-oncogenic role in liver tumors through the regulation of one of the major oncogenes involved in the development and progression of HCC, Ras [11]. Moreover, LKB1 in HCC regulates the Akt-survival pathway independently of phosphoinositide 3-kinase (PI3K) and mammalian target of rapamycin (mTOR) [12]. On the other hand, Akt hyperactivation can contribute to the genesis of cancer coupled to its role in energy metabolism, including the so-called “Warburg effect”. The Akt-dependent changes in energy metabolism in tumor cells involve a coordinated regulation in both glycolysis and oxidative phosphorylation [13]. As with LKB1, the deregulation of the PI3K/Akt pathway is extremely relevant in HCC with over one hundred Akt substrates involved in cell survival, proliferation and metabolism [14]. Importantly, LKB1 and Akt are subjected to PTM regulatory events, as ubiquitination [15]. To date, neddylation regulation of LKB1 and Akt has not been described.

Here, we have unraveled that neddylation is specifically enriched in HCC patients with poor outcomes providing a selective advantage to the tumors. In prohibitin 1 (*Phb1*)-KO mice, neddylation inhibition correlates with a strong modulation of the cancer metabolic signature and liver tumor regression, as a consequence of tumor cell death and characterized by: a) reduced levels of total sphingomyelines (SM) and a variety of diacylglycerols (DAG), succinate, GTP, malonyl-CoA, glycine; and b) augmented glycolic acid, triacylglycerols (TAG) levels and the phosphatiylcholines (PC) synthesis phosphatidylethanolamine methyltransferase (PEMT) flux. In protumoral hepatocytes, neddylation inhibition resulted in metabolic reprogramming rendering a decrease in the oxidative phosphorylation and increased glycolysis, associated with cell apoptosis. Importantly, we found that LKB1 and Akt are new targets of neddylation, undoubtedly providing important insights into the regulatory mechanism of these oncokinases and reinforcing the role of neddylation as a tumor promoter. Evidences of neddylation-mediated driven malignancy further implicate this PTM as a potential novel therapeutic target during liver tumor management and treatment.

## RESULTS

### Alteration of neddylation levels is associated with liver malignancy in clinical HCC

**Figure 1:**
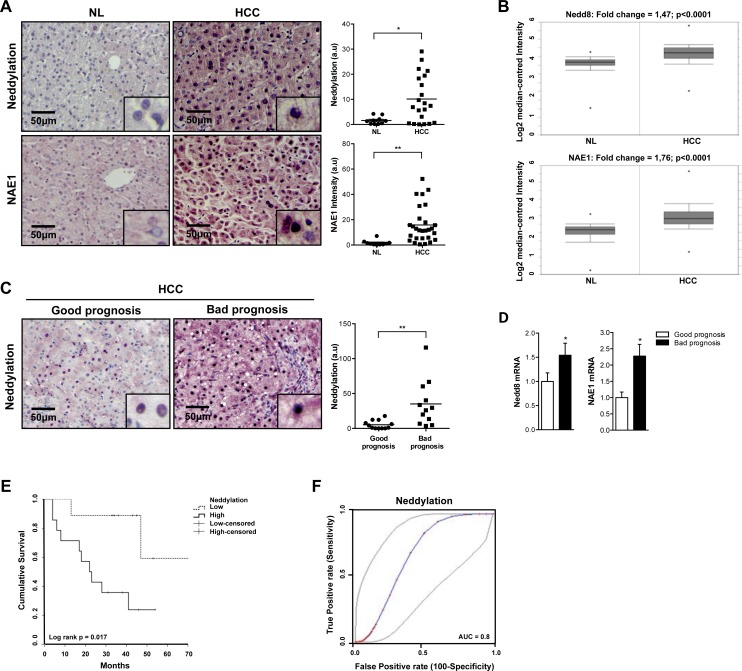
Global neddylation in HCC (A) Nedd8 and NAE1 IHC analysis in human samples from normal liver (NL) (n=10) and hepatocarcinoma (HCC) (n=22) patients. (B) Box plots of Nedd8 and NAE1 gene levels in NL (n=200) and HCC (n=225) human samples from ONCOMINE database. (C) Nedd8 and NAE1 IHC and (D) mRNA expression analysis in good (n=16) and bad (n=13) prognosis HCC human samples. (E) Kaplan-Meier plot of overall survival of HCC patients grouped according to low or high Nedd8 expression. (F) Logistic regression to quantify the predictability of a Nedd8 model in better and poorer prognosis of HCC. Values are represented as mean ± SEM. *p<0.05, **p<0.01 (HCC *vs* NL; Bad *vs* good prognosis).

Dysregulated neddylation pathway was previously described in many types of cancer [16]. Immunohistochemistry (IHC) analyses revealed significantly higher levels of global neddylation and of the Nedd8 activating enzyme E1 (NAE1) in malignant tissues from a cohort of human HCC (Fig. 1A). Additionally, gene expression levels of Nedd8 and NAE1, examined by analysis of published microarrays obtained from an ONCOMINE search (http://www.ncbi.nlm.nih.gov/geo/query/acc.cgi?acc=GSE14520), were increased in a cohort of 225 HCC human samples *versus* 200 controls [17] (Fig. 1B). Neddylation activity increase is more specific of tumoral areas as can be observed in the IHC of a tumor with respective surrounding tissue in Suppl. Fig. 1A. Importantly, in a prospective study of patients with liver cirrhosis on ultrasound surveillance at first diagnosis of HCC, global neddylation at protein level as well as Nedd8 and NAE1 gene expression levels were significantly higher in HCC patients with poor prognosis (Fig. 1C-D). In agreement, Kaplan-Meier plot shows that elevated neddylation levels were associated with lower survival rates (Fig. 1E), while a logistic regression to quantify the predictive value of neddylation revealed an AUC of 0.8 p<0.014 (Fig. 1F).

Overall our data underscore a robust regulation of neddylatio in clinical HCC.

### Neddylation impacts liver cancer metabolism in *Phb1*-KO mice

**Figure 2:**
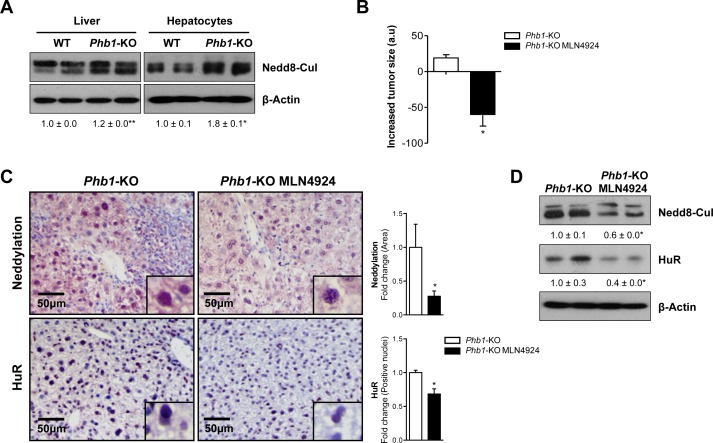
*In vivo* neddylation inhibition blocks tumor progression (A) Representative Western blot analysis of global neddylation (shown as neddylated cullins) in whole extracts from WT and *Phb1*-KO livers and isolated hepatocytes. (B) Reduction of tumor size in *Phb1*-KO mice after MLN4924 treatment determined by caliper measurement of the longest diameter in the transverse view of the liver. (C) Staining of liver sections from *Phb1*-KO MLN4924 treated and *Phb1*-KO untreated mice with Nedd8 and HuR antibodies. Graphical representations are shown on the right of each panel. (D) Western blot analysis of Nedd8 and HuR on liver lysates from *Phb1*-KO MLN4924 and *Phb1*-KO untreated animals. (Values are mean ± SEM. *p<0.05; *Phb1*-KO MLN4924 *vs Phb1*-KO).

To further study neddylation impact on liver cancer, we used *Phb1-*KO mouse model. Prohibitin 1 is a highly conserved, ubiquitously expressed protein that participates in diverse processes including mitochondrial chaperone, growth and apoptosis. Liver-specific deletion of *Phb1* in mice results in spontaneous liver injury, fibrosis, and HCC [18]. Like in clinical HCC, increased neddylation levels, measured as accumulation of Nedd8-cullin conjugated levels, associated with the presence of malignant nodules in livers and hepatocytes from *Phb1 -KO* mice were observed (Fig. 2A, Suppl. Fig. 1B). In agreement, neddylation was induced in human hepatoma cell lines compared to non-tumoral hepatocytes, (Suppl. Fig. 1C), and in other animal models that spontaneously develop HCC, such as the glycine N-methyltransferase *(Gnmt)*-KO mice [19] (Suppl. Fig. 1D). Considering the elevated neddylation levels in the *Phb1*-KO mice, the therapeutic potential of the small molecule neddylation inhibitor, MLN4924, was evaluated. For this purpose, 10 *Phb1*-KO mice, from 5 to 6-months old, were selected and randomly assigned to two different experimental groups: (i) injected subcutaneously with MLN4924 (60 mg/kg body weight) and (ii) vehicle-treated, once a week for 8 weeks. Importantly, longitudinal evaluation by *in vivo* high-frequency ultrasound fortnightly revealed a strong reduction in the liver tumor size in the MLN4924-treated mice (Fig. 2B, Suppl Table I) without visible biochemical side effects as a consequence of the MLN4924-treatment (Suppl. Fig. 2A, Suppl. Table II). Moreover, decreased tumor size in the MLN4924-treated group was associated with a down regulation of global neddylation, observed by decreased Nedd8-cullin conjugates and, of the already known Nedd8 target, HuR [8] (Fig. 2C-D). As expected, neddylation inhibition with MLN4924 promoted an upregulation of the cullin-targets c-Jun and Nrf2 (Suppl. Fig. 2B), as detected by Western blot analysis. In addition, neddylation inhibition in *Phb1*-KO mice reduced liver fibrosis (Suppl. Fig. 2A), further supporting the beneficial role of MLN4924-treatment without the appearance of secondary complications. High-throughput metabolomic analysis on liver samples was performed to highlight some of the mechanisms underlying tumor regression as a consequence of neddylation inhibition.

Wide targeted metabolomics data revealed a strong reduction of sphigomyelins (SM), SM(d18:0/22:0) (90%, p<0.05) together with decreased diacylglycerols (DAG) levels [DAG(32:1), DAG(32:2), DAG(34:2)] (~30% p<0.05) in MLN4924-treated *Phb1*-KO mice (Suppl. Table III, and Suppl Fig. 2C). On other hand, triacylglycerols (TAG) were found to be increased as a result of neddylation inhibition [TAG(54:6), TAG(54:7), TAG(56:7), TAG(56:8), TAG(58:10), TAG(58:9)]. Importantly, MLN4924 treatment increased the hepatic levels of some types of phosphatidylcholines (PC) [PC(18:0/20:4) (69% p< 0.05), PC(18:0/22:6) (89% p<0.05)] and reduced those of the phosphatidylethanolamines (PE) [PE(16:0/18:1) (54% p<0.05)] contributing to a significant shift (40%, p<0.05) in the 16:0/18:1 and 18:0/20:4 species PC/PE ratio (Suppl. Table III). In the liver, PC can be formed either by the Kennedy pathway or by the phosphatidylethanolamine *N*-methyltransferases (PEMT) pathway. Phosphatidylcholines formed via PEMT pathway are primarily enriched in long-chain polyunsaturated fatty acids (PUFA), such as docosahexaenoic acid (22:6n-3) [20]. Thus, an increase in the PC(22:6n-3)/total PC ratio (20%, p<0.05) was identified in MLN4924-treated *Phb1*-KO mice suggesting increased PEMT flux.

Moreover, we observed that the central carbon metabolism was tightly controlled by neddylation inhibition in *Phb1*-KO mouse (Suppl. Table IV, Suppl. Fig. 2D). Importantly, pivotal metabolites like succinate [21, 22] were reduced after MLN4924-treatment. In agreement, GTP levels, a readout metabolite from the catabolism of succinate mediated by succinyl-CoA synthetase, were diminished under these conditions. In addition, the hepatic content of malonyl-CoA, a classical β-oxidation inhibitor, was also downregulated in mice upon MLN4924 treatment (Suppl. Table IV, Suppl. Fig. 1D). Importantly, glycine, a metabolite that reflects proliferation rate across cancer cells [23], was attenuated (21%, p< 0.05) after NAE1 inhibition. Finally, glycolic acid, the classical inhibitor of the aerobic glycolysis through lactate dehydrogenase (LDH) activity [24], was increased in the liver of MLN4924-treated *Phb1*-KO mice.

Overall, our data underscore that neddylation inhibition as a result of MLN4924 treatment to *Phb1*-KO mice is associated with tumor regression associated with metabolic reprogramming.

### Neddylation inhibition promotes apoptosis in *Phb1*-KO hepatocytes associated with a metabolic shift from oxidative phosphorylation to glycolysis

**Figure 3:**
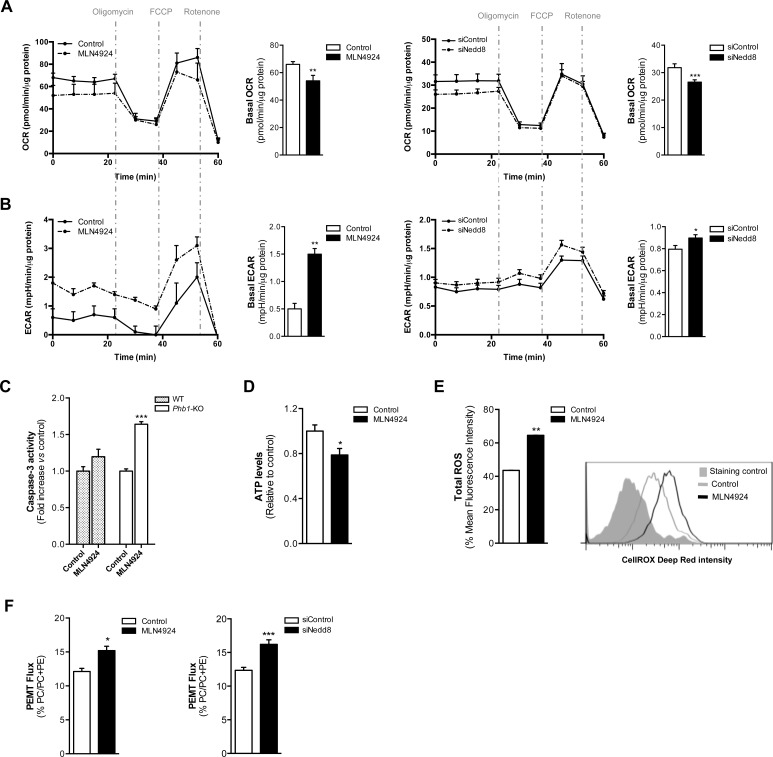
Oxidative Phosphorylation and Glycolysis are dependent on neddylation activity (A) Oxygen consumption rate (OCR) and (B) extracellular acidification rate (ECAR) values in *Phb1*-KO hepatocytes 48 hours after MLN4924 treatment or Nedd8 silencing. The energetic response in cells was measured in the presence of oligomycin, FCCP and rotenone. (C) Caspase-3 activity in WT and *Phb1*-KO hepatocytes after 48 hours of MLN4924 treatment. (D) ATP levels and (E) ROS production in *Phb1*-KO hepatocytes after 48 hours of MLN4924 treatment. (F) Phosphatidylethanolamine methyltransferase (PEMT) flux in *Phb1*-KO hepatocytes 48 hours after MLN4924 treatment or Nedd8 silencing. Values are represented as mean ± SEM. n=4 samples/time point. *p<0.05, **p<0.01, ***p<0.001 (MLN4924 or siNedd8 *vs* control).

To further investigate the role of neddylation loss on tumor cell metabolism, we examined energetic metabolism using a Seahorse^®^ XF24 Analyzer equipment in hepatocytes isolated from *Phb1-*KO mice. For this purpose, hepatocytes were harvested from 8-month old animals in order to obtain well-differentiated hepatocytes showing a protumoral phenotype. The cells upon neddylation inhibition, both with MLN4924 and Nedd8 silencing, displayed a reduction in the oxygen consumption rate (OCR), a surrogate of oxidative phosphorylation (OXPHOS) (Fig. 3A). Moreover, the extracellular acidification rate (ECAR), a measure of glycolysis, was increased as a result of neddylation reduction (Fig. 3B). The observed OXPHOS reduction and ECAR induction resultant of neddylation inhibition were associated with an apoptotic response as analyzed by caspase-3 activity (Fig 3C). Importantly, apoptosis induction by MLN4924 treatment was only observed in *Phb1*-KO hepatocytes and not in healthy hepatocytes from wild type animals. Moreover, MLN4924 induced cell death in *Phb1*-KO hepatocytes was associated with a reduction in ATP (Fig. 3D) and augmented redox levels (Fig. 3E).

Noteworthy, neddylation inhibition, using either MLN4924 or Nedd8 siRNA, led to a strong stimulation in the PEMT flux as shown for the incorporation of [^3^H] ethanolamine into PE and PC in *Phb1*-KO hepatocytes (Fig. 3F). The observed PEMT pathway induction as a consequence of neddylation inhibition was observed both following *in vivo* treatment as well as in isolated hepatocytes. Previous work has shown that MLN4924 treatment induces apoptosis in hepatoma cells [25]. Here, we have extended this analysis and we have shown also that apoptosis mediated by neddylation inhibition induced either by MLN4924 or by Nedd8 silencing, in human hepatoma cells, is accompanied by a decrease in OXPHOS, an increase in the glycolytic flux and a higher activity of the PEMT pathway (Suppl. Fig 3A-E).

All together our data support that neddylation inhibition-induced apoptosis in *Phb1*-KO hepatocytes is accompanied by alterations in cell metabolism as decreased OXPHOS, increased glycolytic flux and higher activity of the PEMT pathway.

### New neddylation targets identified in liver cancer

**Figure 4:**
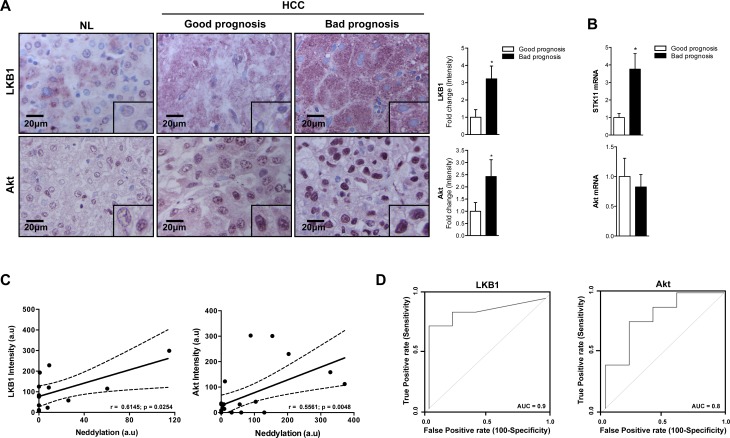
Association between LKB1, Akt and neddylation in human HCC (A) LKB1 and Akt IHC analysis in human samples from normal liver (NL) and HCC human samples from patients with good and bad prognosis. Graphical representations are shown on the right of each panel. (B) LKB1 and Akt mRNA expression in human samples from patients with HCC with good or bad prognosis. (C) Pearson's correlation between neddylation and LKB1 or Akt levels respectively. (D) ROC curves of LKB1 and Akt in good and bad prognosis of human HCC samples. The area under the ROC curve (AUC) measures the statistical potential of Akt and LKB1 to differentiate the two prognosis groups. Values are represented as mean ± SEM. *p<0.05 (Bad *vs* good prognosis).

Many of the oncogenic drivers observed in cancer alter tumor-metabolism as part of their mode of action. LKB1 and Akt are well known metabolism regulators in healthy liver. However, the activity of both kinases has been associated with HCC development [11-14]. Importantly, IHC and Western blot analysis showed that LKB1 and Akt levels were considerably overexpressed in HCC patients, being further increased in those with poor outcome prognosis (Fig. 4A). Whereas increased protein levels of LKB1 are associated with the transcriptional upregulation of *STK11* (the gene encoding for LKB1), the same is not valid for Akt (Fig. 4B).

Notably, the logistic regression model showed a good predictability for the levels of LKB1, Akt and global neddylation (Fig. 4C) suggesting that the increased levels of LKB1 and Akt in patients with liver cancer is associated to elevated hepatic neddylation. In addition, LKB1 and Akt ROC curves provide a significant value of area under the curve (AUC) close to 1, indicating the good overall ability of the test to discriminate between good and bad prognosis (Fig. 4D).

**Figure 5:**
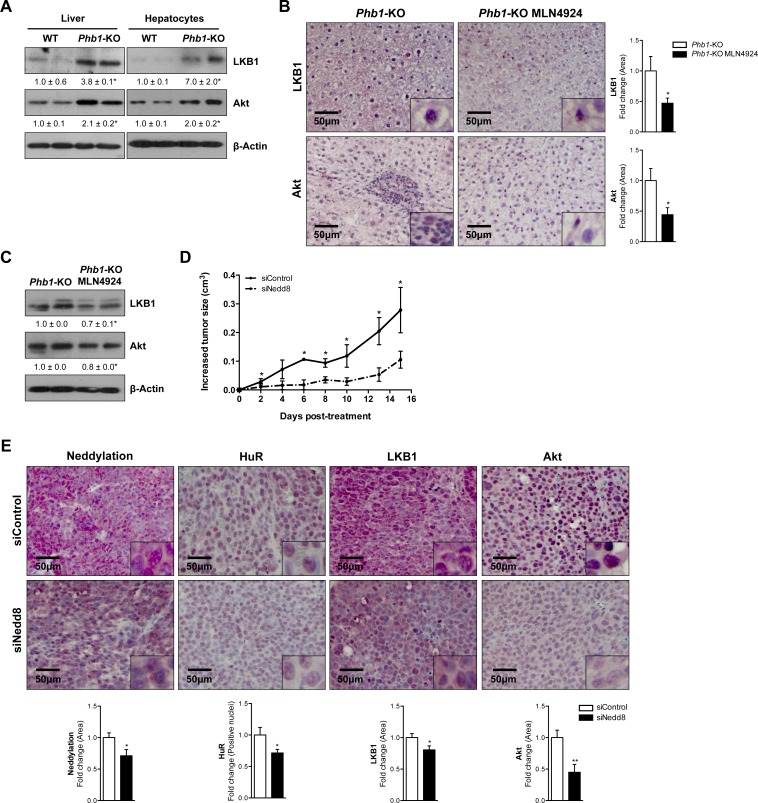
LKB1 and Akt levels are dependent of neddylation in HCC mice models (A) Representative Western blot analysis of LKB1 and Akt in whole extracts from WT and *Phb1*-KO livers and isolated hepatocytes. (B) Staining of liver sections from *Phb1*-KO MLN4924 treated and *Phb1*-KO untreated mice with LKB1 and Akt antibodies. Graphical representations are shown on the right of each panel. (C) Western blot analysis of LKB1 and Akt on liver lysates from *Phb1*-KO MLN4924 and *Phb1*-KO untreated animals. (D) Graphical representation of changes in tumor size in HepG2-xenografted nude mice. (E) Staining and graphical representation of quantitative analysis of each staining in HepG2-xenografted nude mice tumors. Values are represented as mean ± SEM. *p<0.05, **p<0.01 (*Phb1*-KO MLN4924 *vs Phb1*-KO; siNedd8 *vs* siControl).

As observed in clinical HCC, LKB1 and Akt levels were also significantly overexpressed in the liver and hepatocytes from *Phb1-*KO mice (Fig 5A), although these proteins transcriptional levels were not significantly altered (Suppl. Fig. 4A). Importantly*,* the neddylation inhibition as a result of MLN4924-treatment was correlated with a reduction of the LKB1 and Akt oncogenic proteins (Fig. 5B-C) only at the protein level (Suppl. Fig. 4B). LKB1 is an upstream regulator of AMPK. However, treatment with MLN4924 was not associated with decreased AMPK activation (Suppl. Fig. 2E), suggesting an uncoupling between LKB1 and AMPK under neddylation inhibition in liver tumor models. Increased phosphorylation of AMPK under neddylation inhibition correlated with increased caspase-3 as observed earlier [11].

**Figure 6:**
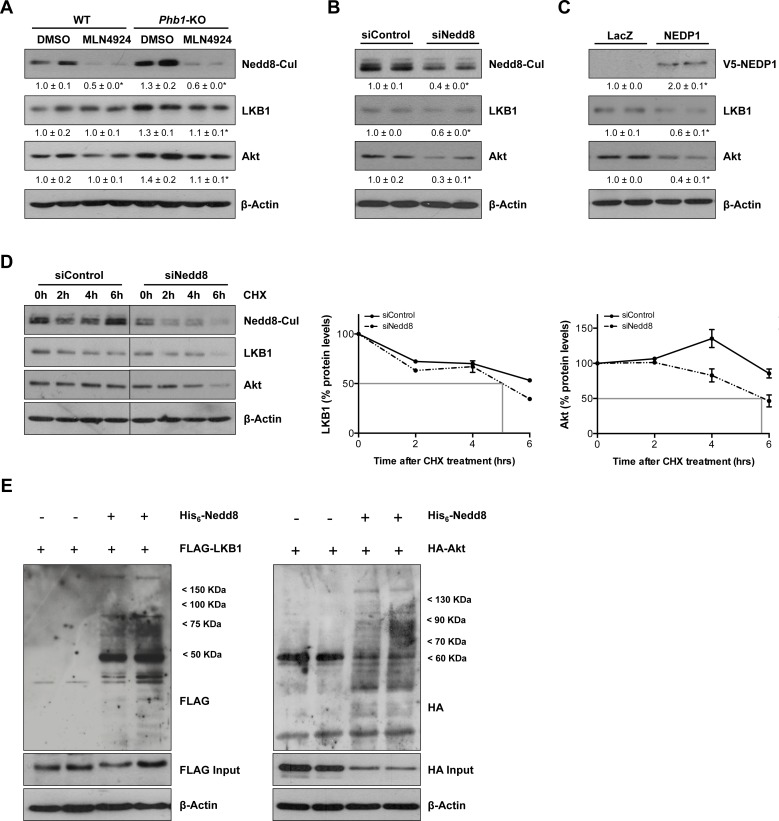
LKB1 and Akt as new targets of neddylation (A) Representative Western blot analysis of total lysates from WT and *Phb1*-KO hepatocytes treated with MLN4924 for 48 hours using Nedd8, LKB1 and Akt antibodies. LKB1 and Akt levels were also evaluated by Western blot in *Phb1*-KO hepatocytes after (B) Nedd8 silencing and (C) NEDP1-V5 overexpression. (D) The stability of LKB1 and Akt proteins as the percentage of protein level remaining after treated with cycloheximide (CHX). Vertical bars are indicative of the calculated half-life for every protein. (E) FLAG and HA immunoreactive proteins in WT hepatocytes expressing FLAG-LKB1 or HA-Akt and His_6_-Nedd8. Hepatocytes were cotransfected with FLAG-LKB1/HA-Akt and His_6_-Nedd8 plasmids, and FLAG/HA-containing proteins were purified using Ni^2+^-NTA and detected by Western blotting.

Furthermore, we evaluated the effect of the *in vivo* Nedd8 silencing, using siRNA approaches, in the well-established *in vivo* tumorigenesis HepG2 xenograft mouse model. In these animals, a 66±11% decrease (p<0.05) in tumor size (Fig. 5D) and a reduction of the levels of HuR observed as a consequence of neddylation inhibition, were also associated with decreased LKB1 and Akt levels (Fig. 5E). As detected *in vivo*, neddylation inhibition, both after MLN4924-treatment and knockdown of Nedd8 by siRNA in *Phb1-*KO isolated hepatocytes was also accompanied with a fall in the total amount of the oncogenic proteins, LKB1 and Akt (Fig. 6A-B), without regulation at the transcriptional level (Suppl. Fig. 4C). In addition, Nedd8 specific-protease cysteine (NEDP1) overexpression, which specifically removes Nedd8 molecules from conjugated substrates [26], also reduced LKB1 and Akt levels in *Phb1*-KO hepatocytes (Fig. 6C), without affecting their transcriptional regulatioh (Suppl. Fig. 4C). As expected, Nedd8-silencing accounts for decreased stability of LKB1 and Akt, as observed from the reduced half-life of these proteins after the inhibition of protein synthesis by cycloheximide (Fig. 6D). The correlation found between global neddylation and LKB1 and Akt levels, prompted us to hypothesize that LKB1 and Akt could be in partly regulated through neddylation. To verify this, we overexpressed His_6_-Nedd8 in hepatocytes from wild type animals. The enrichment analysis of His_6_-Nedd8 conjugated proteins revealed enhanced molecular bands of LKB1 and Akt (Fig. 6E).

In summary, we reveal for the first time that LKB1 and Akt are direct neddylation targets. As such, neddylation inhibition is able to impair LKB1 and Akt stabilization contributing to regulate the levels of these two oncokinases.

### LKB1 and Akt stabilization drives the metabolic reprogramming induced by neddylation inhibition

**Figure 7:**
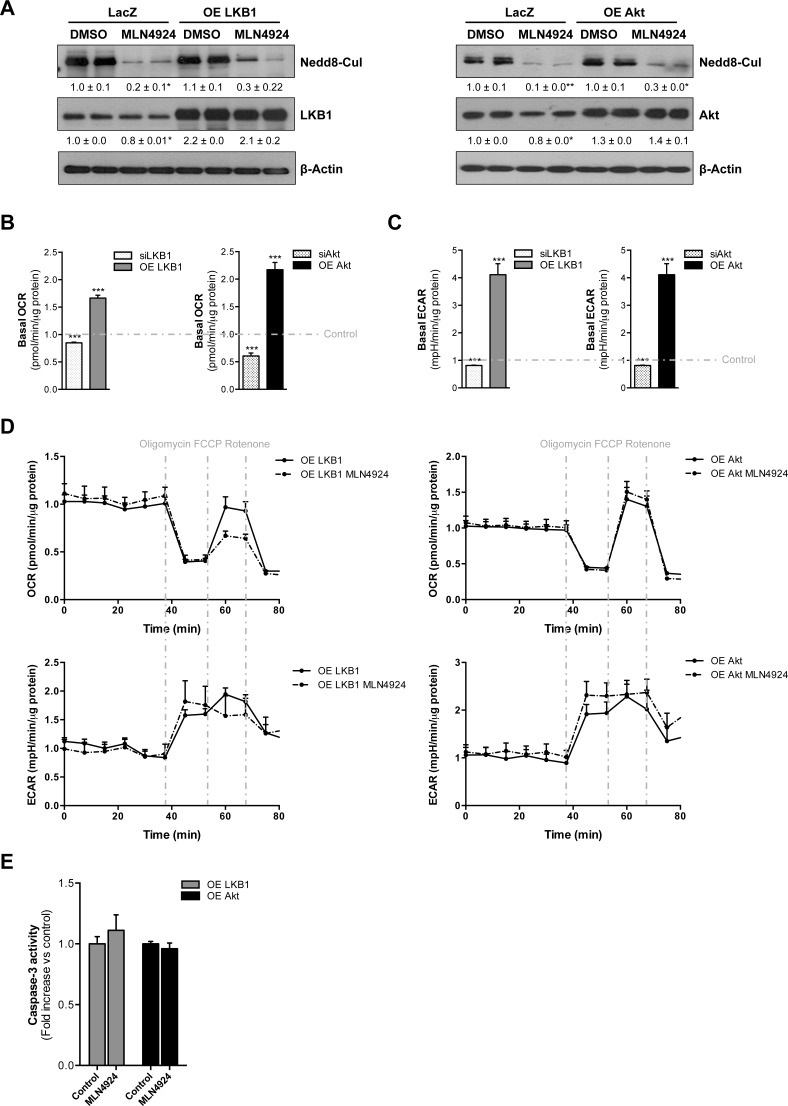
LKB1 and Akt stabilization play an important role on neddylation-induced metabolic disruptions in liver cancer (A) Western blot analysis against Nedd8, LKB1 and Akt in *Phb1*-KO hepatocytes overexpressing Akt and LKB1 after 48 hours of MLN4924 treatment. (B) Basal oxygen consumption rate (OCR) and (C) extracellular acidification rate (ECAR) values in *Phb1*-KO hepatocytes after LKB1 and Akt overexpression and silencing. (D) OCR and ECAR were measured in *Phb1*-KO hepatocytes overexpressing LKB1 and Akt after 48 hours of MLN4924 treatment. The energetic response in cells was measured in the presence of oligomycin, FCCP and rotenone. (E) Caspase-3 activity in *Phb1*-KO hepatocytes overexpressing LKB1 and Akt after 48 hours of MLN4924 treatment. Values are mean ± SEM. *p<0.5, ***p<0.001 (LKB1/Akt overexpression and silencing *vs* control).

To investigate whether the neddylation inhibition induced-apoptosis in *Phb1*-KO isolated hepatocytes was partially mediated by the decrease in LKB1 and Akt protein stabilization, we overexpressed these two kinases under MLN4924 treatment (Fig. 7A). Overexpression of both LKB1 and Akt *per se* accounts for increased OCR and ECAR thereby promoting a more proliferative phenotype in these premalignant hepatocytes (Fig 7B-C). On the other hand, LKB1 and Akt silencing (Suppl. Fig. 4D) was associated with decreased OCR and ECAR (Fig. 7B-C). Moreover, MLN4924 treatment under LKB1 and Akt-overexpression did not significantly alter OCR and ECAR levels (Fig 7D). Interestingly, boosting the levels of both LKB1 and Akt was sufficient to counteract the apoptotic response mediated by neddylation inhibition (Fig 7E).

In summary, the restoration of LKB1 and Akt expression was able to block the metabolic reprogramming and associated cell apoptosis induced by neddylation inhibition. These data further support that both LKB1 and Akt destabilization partially drive the metabolic phenotype induced by lack of neddylation.

## DISCUSSION

Multiple signaling pathways appear to converge in HCC [1]. The current view of cancer progression highlights that cancer cells must undergo through a PTM regulation and metabolic reprogramming in order to progress in an unfriendly environment. Under this basis, the neddylation pathway is essential for cancer development through the activation of Cullin ring-ligases and the degradation of their substrates [4]. In addition, the neddylation-induced stabilization of other target proteins can occur. Herein, we have investigated the importance of neddylation in liver cancer signaling and metabolism and more importantly its potential as a new therapeutical approach.

We provide strong evidences that high neddylation is associated with clinical HCC and poorest prognosis, suggesting that it provides an advantage to tumor cells. Moreover, in *Phb1*-KO HCC mice, reduced neddylation levels as a result of its pharmacological inhibition using the small molecule inhibitor MLN4924 was associated with a distinguishable metabolic fingerprint associated with tumor regression and characterized by: a) reduced levels of total SM and variety of DAG, succinate, GTP, malonyl-CoA, glycine; and b) augmented glycolic acid, TAG levels and PEMT flux.

Recently, SM has emerged as an important molecule to generate bioactive sphingolipids through ceramides. SM synthase is an enzyme that generates SM and DAG from PC and ceramides. As such, reduced SM and DAG levels could reflect decreased SM synthase activity, which is inhibited when the hepatocytes enter into the S-phase after partial hepatectomy [27]. Based on these evidences our results clearly suggest an association between SM synthase, with reduced SM and DAG levels and diminished proliferative phenotype. Moreover, DAG cellular content has been previously associated with oxidative stress, redox misbalance and mitochondrial dysfunction [28]. Also, DAG exhibits a dual role in cell biology serving as substrate for cell membrane biosynthesis (phospholipids) and energy storage as TAG. Thereby, under neddylation inhibition conditions, reduced DAG levels as a result of improved mitochondrial function and decreased proliferation may be channeled to TAG synthesis. Lipidomics and enzymatic analysis showed that neddylation inhibition resulted in augmented PEMT activity in *Phb1*-KO mice. These data are in agreement with the potential role of PEMT as a liver-specific tumor suppressor and the inhibitory effects exerted by the PEMT-dependent PC species in the Kennedy pathway required for hepatocyte proliferation [29, 30]. Targeted metabolomics performed in regressive liver tumors of *Phb1*-KO mice after neddylation inhibition revealed an improvement in TCA cycle and mitochondrial functions as shown by a reduction in succinate, GTP and glycine levels, metabolites often described as pro-proliferative and showing hepatic damage-inducing properties [21-23]. Moreover, reduced malonyl-CoA levels account for augmented β-oxidation and formation of acetyl-CoA to enter and further sustain restored TCA cycle flux. As a result of improved mitochondrial function, aerobic glycolysis is reduced as shown by augmented glycolic acid levels, an inhibitor of lactate dehydrogenase activity [24]. In summary, following neddylation inhibition in *Phb1*-KO mice, a reduced malignant phenotype translating into tumor regression as a consequence of tumor cell death is observed.

In the last couple of years, neddylation inhibition has been widely implicated in tumor cell death in several tissues [16]. In order to further study neddylation inhibition-induced tumor cell death, we used well-differentiated tumor *Phb1*-KO hepatocytes. Herein, the metabolic slowdown observed after neddylation inhibition was due to the negative impact that the absence of Nedd8 has in the OXPHOS pathway ending in cancer cell apoptosis. The mitochondrial apoptosis induced by neddylation inhibition was also associated with an increase of the PEMT flux as previously observed [31]. These results are in agreement with early evidences implicating MLN4924 and thereby neddylation inhibition actions on cellular redox dysfunction [32]. As a consequence of decreased OXPHOS, tumor cells need to look for alternative energetic pathways such as glycolysis thereby resulting in increased glycolytic flux. Interestingly, the metabolic switch from OXPHOS to glycolytic flux, usually advantageous to tumor cells, in here is associated with increased tumor cell apoptosis. We speculate that this is due to the fact that under neddylation inhibition the cellular energetic pool, already compromised by reduced OXPHOS, is being channeled to the high-demanding energetic process of DNA rereplication, previously shown to be induced in neddylation inhibition [33]. Importantly, a reduction in the OXPHOS/glycolysis metabolic ratio as a consequence of neddylation inhibition is associated with augmented tumor cell apoptosis.

Considering that LKB1 and Akt kinases play an important role in energetic metabolism regulation and have been previously shown to be targets of PTM regulatory events, we hypothesized that LKB1 and Akt are potential neddylation targets. In agreement, during the transformation of primary hepatocytes, in the liver of *Phb1-*KO HCC mice and more importantly in a cohort of human HCC samples, a strong correlation in the levels of LKB1, Akt and Nedd8 in the liver was observed. In here, we report for the first time that both LKB1 and Akt are new targets of neddylation modifications. Indeed the overexpression of either LKB1 or Akt kinases counteracts the metabolic reprogramming response mediated by neddylation inhibition associated to mitochondrial apoptosis (Suppl. Fig. 5). This is in agreement with early reports showing that LKB1 deficient cells are less able to restore ATP levels in response to metabolic stress and thus, more susceptible to cell death [34], and with the important role of Akt in OXPHOS and glycolysis balance [35].

Summing up, our results reveal for the first time a completely new and unexplored role of neddylation in the stabilization of the key oncogenic hallmarks, LKB1 and Akt. Moreover, we provide evidences that LKB1 and Akt stabilization plays an important role on neddylation-induced metabolic disruptions in liver cancer further suggesting neddylation as a potential therapeutic target in HCC.

## MATERIALS AND METHODS

### Human Samples

Surgically resected liver specimens of 22 patients with HCC (10 Hepatitis C, 10 ASH and 2 NASH) were examined. The Basque Research Biobank (http://www.biobasque.org) provided the data and type of biospecimen. For the bad and better prognosis analyses we used samples from 47 patients with liver cirrhosis and HCC detected during surveillance. They had preserved liver function and corresponded to BCLC stage A (n=34) and B (n=13). These patients are part of another study, reported elsewhere (Gut, submitted). All tissues samples from this study were from US-guided liver biopsy of HCC lesions. In brief, compensated patients with liver cirrhosis under ultrasonographic surveillance for HCC, when first identified as having developed a HCC lesion underwent a dedicated imaging protocol (2 computed tomography exams 6-weeks apart in a absence of any other therapy to evaluate growth speed) and an US-guided liver biopsy (to define transcriptomic characteristics of tumor). After the 2^nd^ computed scan, patients underwent therapy according to internationally accepted guidelines. Patients were divided in 4 quartiles according to HCC volume doubling time and survival evaluated according to these quartiles. Patients of the first quartile (Doubling time< 53 days) had a definitively worse prognosis that those of the other quartiles (median survival 11 months *vs* 41, 42 and 47 months, respectively). Healthy human liver was used as control for immunostaining. Patients gave informed consent to all clinical investigations, in according to the principles embodied in the Declaration of Helsinki.

### Animals

Three to eight month-old male *Phb1*-KO mice [18], breded in the animal facility at the CIC bioGUNE, were used. Animal procedures were approved following the CIC bioGUNE Animal Facility's guidelines with AALAC certificate.

### Cell Lines

*In vitro* experiments were performed using primary mouse hepatocytes from *Phb1*-KO and the human hepatoma cell lines BCLC3 and BCLC5, characterized and provided by Dr. Bruix and Dr. Boix (BCLC group. Hospital Clinic, Barcelona, Spain).

### Isolation and Culture of Primary Hepatocytes

Primary hepatocytes were isolated from male C57BL6 WT and *Phb1*-KO mice via collagenase perfusion as described before [8].

### Cell Treatment with MLN4924

MLN4924 was provided by Millenium Pahrmaceuticals Inc. *Phb1*-KO hepatocytes were treated with MLN4924 at the dose of 3 μM for 48 hours using DMSO as control.

### Cell Transfection

*Phb1*-KO hepatocytes were transfected with 2 μg of pcDNA3-FLAG-LKB1 (Addgene), pLNCX1-HA-Akt (kindly provided by Dr. Carracedo, CIC bioGUNE, Spain) or cysteine protease (NEDP1-V5) plasmids using jetPRIME^TM^ reagent (Polyplus). Two μg of pcDNA3-LacZ (Invitrogen) and pLNCX1 plasmids (provided by Dr. Carracedo) were used as negative controls of LKB1 and Akt, respectively.

### *In vitro* Silencing

Primary *Phb1*-KO hepatocytes were transfected with 100 nM Nedd8, LKB1 and Akt siRNA (Qiagen) using jetPRIME^TM^ reagent (Polyplus). Controls were transfected with an unrelated siRNA (Qiagen). Protein knockdown was confirmed by Western blotting. siRNA sequences are shown in Supplementary Table V.

### *In vivo* MLN4924 Treatment

MLN4924 was injected subcutaneously into 10 *Phb1*-KO mice, once a week at a dose of 60 mg/kg for 8 weeks, following the Millennium Corporation communication. Age-matched *Phb1*-KO controls, corresponding to the untreated group, were injected with the respective vehicle at the same time intervals.

### *In vivo* Nedd8 silencing

5×10^6^ HepG2 cells were injected into 10 male athymic C57BL/6J nude mice, as described in Supplemental Material. One week after cell inoculation, animals were divided into 2 experimental groups: (i) siControl (n=5) and (ii) siNedd8 (n=5), 50 μM-siRNA dose were intraperitoneally injected thrice a week using jetPEI^TM^ reagent (Polyplus), following manufacturer's instructions.

### Immunohistochemistry

Paraffin embedded liver samples were sectioned, dewaxed and hydrated. All procedures were done according to standard protocols with EnVision+ System HRP (Dako). Finally, samples were incubated with Vector Vip substrate (Vector) for purple color development. 10 random images per sample were taken with a 10x objective from a microscope AXIO Imager D1 (Carl Zeiss AG, Oberkochen, Germany). Quantification of staining intensity or area of each sample were calculated using the manual selection tool of FRIDA software (FRamework for Image Dataset Analysis) http://bui3.win.ad.jhu.edu/frida/. Stained area was measured using ImageJ64 software (NIH), images were transformed to RGB stacks, manually thresholded for selection of the stained área and measured with the measure tool to calculate the area percentage. A description of the antibodies used is provided in Supplementary Table VI.

### Protein Isolation & Western Blotting

Extraction of total protein from cultured cells and livers was performed as described [8]. 4 to 25μg of protein were electrophoresed on sodium dodecyl sulfate-polyacrylamide gels and transferred onto membranes. A description of the antibodies used is provided in Supplementary Table VI. Band intensities were quantified using the ImageJ software and normalized to the β-Actin housekeeping. All experiments were performed at least 5 times.

### RNA Isolation and Quantitative Real-Time PCR

RNA was isolated with Trizol (Invitrogen), and its concentration and integrity were determined. PCRs were performed using iQ™ SYBR^®^ Green Supermix (Biorad) and the Bio-Rad iCycler thermocycler (Bio-Rad, Hercules, CA). The Ct values were extrapolated to a standard curve, and data was then normalized to the house-keeping expression (18S and GAPDH). The sequences of primers used are described in Supplementary Table VII.

### Apoptosis Measurement

Caspase-3 activity was measured as previously described [8].

### ATP Levels

ATP levels were measured by using the commercial available kit ATPlite^TM^ (Perkin Elmer).

### Evaluation of ROS production

ROS production was measured by flow cytometry analysis using CellROX^TM^ Deep Red according to manufacters intructions (Life Technologies).

### Cycloheximide Treatment

*Phb1*-KO hepatocytes were transfected with Control or Nedd8 siRNA as described. Later, cycloheximide (CHX; 50 μg/mL) was added, and, at the indicated times, cells were lysed. Protein was analyzed by Western blotting using the indicated antibodies, quantified with Image J software, and presented as the percentage of remaining protein. Data are representative from three independent experiments.

### Protein-Histidine Affinity Purification Using Nickel-Nitrilotriacetic Acid Beads

*Phb1*-KO hepatocytes were cotransfected with 2μg of His_6_-Nedd8 and FLAG-LKB1 or HA-Akt constructs using jetPRIME^TM^ reagent (Polyplus). Cells were lysed in 6 M guanidinium-HCl, 0.1 M Na_2_HPO_4_/NaH_2_PO_4_, 0.01 M Tris-HCl pH 8, plus 10 mM β-mercaptoethanol and 5mM Imidazole. Lysates were mixed with 70 μl of low density Ni^2+^-NTA-agarose beads (ABT) precoated with BSA and prewashed with lysis buffer. Lysates were incubated with the beads for 3 hours at RT, successively washed first with lysis buffer, then twice with 8 M urea, 0.1 M Na_2_HPO_4_/NaH_2_PO_4_, 0.01 M Tris-HCl pH 8 plus 10 mM β-mercaptoethanol, and finally thrice with 8 M urea, 0.1 M Na_2_HPO_4_/NaH_2_PO_4_, 0.01 M Tris-HCl pH 6.3 plus 10 mM β-mercaptoethanol. After last wash, the beads were eluted with 200 mM Imidazole in 5% SDS, 0.15 M Tris-HCl pH 6.7, 30% glycerol, 0.72 M β-mercaptoethanol. The eluates were subjected to SDS-PAGE and the proteins transferred to a nitrocellulose membrane for Western blotting against FLAG (Sigma) and HA (Covance) antibodies.

### *In vivo* Ultrasound Imaging

The Vevo 770 high-frequency Ultrasound (US) system (VisualSonics Inc., Toronto, Ontario, Canada) was used employing a 40-MHz probe (Scanhead RMV-704, VisualSonics Inc., Toronto, Ontario, Canada). Further information is provided in Supplementary Material.

### Measurements of oxygen consumption rate (OCR) and extracellular acidification rate (ECAR)

A Seahorse Bioscience XF24-3 Extracellular Flux Analyzer was used to measure the rate change of dissolved O_2_ and pH in medium immediately surrounding adherent cells. Further information is provided in Supplementary Material.

### Quantification of Phosphatidylethanolamine and Phosphatidylcholine Synthesis Rates using Radioisotopes

Hepatocytes were incubated with [^3^H]ethanolamine (5 μCi/ml). Cells and medium were separated, lipids extracted, separated, and the label incorporated into phosphatidylethanolamine (PE) and phosphatidylcholine (PC) determined in a scintillation counter.

### Metabolomics Analysis

UPLC^®^-MS metabolomics analysis was performed in livers from untreated *Phb1*-KO and MLN4924 treated mice. The specific procedures, such as metabolite extraction, data processing, normalization and statistical analysis have been previously detailed and are described extensively in Supplementary Material.

### Statistical Analysis

All experiments were performed at least in triplicate. Data are expressed as mean ± SEM. Statistical significance was estimated with Student's t test. A *p* value < 0.05 was considered significant.

## SUPPLEMENTARY MATERIAL FIGURES AND TABLES



## Abbreviations

AMPK: AMP-activated protein kinase
BCLC: Barcelona Clinic Liver Cancer
DAG: Diacylglycerols
ECAR: Extracellular acidification rate
HCC: Hepatocellular carcinoma
HuR: Human antigen R
LDH: Lactate dehydrogenase
LKB1: Liver Kinase B1
NAE1: Nedd8 activating enzyme
Nedd8: Neural precursor cell expressed developmentally down-regulated 8
NEDP1: Nedd8 specific-protease cysteine
OCR: Oxygen consumption rate
OXPHOS: Oxidative phosphorylation
PC: Phosphatidylcholine
PE: Posphatidylethanolamine
PEMT: Phosphatidylethanolamine methyltransferase
*Phb1*-KO: Prohibitin1-Knockout
PI3K: Phosphoinositide 3-kinase
PTM: Post-traslational modification
PUFA: Polyunsaturated fatty acids
SM: Sphingomyelins
TAG: Triacylglycerols
TCA: Tricarboxylic acid
