# Supplementary material for: Stabilization of LKB1 and Akt by neddylation regulates energy metabolism in liver cancer
Source: Oncotarget. 2014 Dec 11;6(4):2509–23. doi: 10.18632/oncotarget.3191 (PMC4385867; doi:10.18632/oncotarget.3191)
Supplement: Supplementary file 1 [file oncotarget-06-2509-s001.pdf]

Stabilization of LKB1 and Akt by neddylation regulates energy metabolism in liver cancer

SUPPLEMENTARY MATERIAL

HepG2 xenograft model. The HepG2 xenograft model was established by subcutaneous injection of 5×10^6 HepG2 cells at the right flank of 10 male athymic C57BL/6J nude mice. Cells were cultured in Dulbecco's Eagle's minimum essential medium (DMEM) supplemented with 10% fetal bovine serum and maintained under a 5% CO_2-humidified atmosphere at 37°C. Cells were trypsinized and resuspended in 100µl of a DMEM/matrigel solution (70µl DMEM and 30µl matrigel) for injection. One week after cell inoculation, when the tumors were palpable, animals were divided into two experimental groups: (i) siControl (n=5) and (ii) SiNedd8 (n=5). µ50M-siRNA dose were intraperitoneally injected using jetPEI (Polyplus) three times per week. All animal manipulations were performed under sterile conditions.

Determination of tumor growth. Tumor volume was measured thrice a week during the treatment and prior to necropsy. Tumor volumes were estimated with a caliper and calculated according to the following formula: Tumor volume (cm^3)=(length [mm]×width^2 [cm^2])/2.

***In vivo* Ultrasound Imaging**. During imaging sessions, mice were kept under anesthesia with 1.5% isoflurane in oxygen and restrained on a heated stage (THM-150, Indus Instruments, Houston, TX). Images of the liver were acquired through the ventral body wall in transverse and sagittal orientations in each animal fortnightly during 8 weeks. The liver parenchyma was examined for echogenicity, homogeneity, presence or absence of nodules, echostructure and border definition. The size of the nodules was determined by caliper measurement of the longest diameter in the transverse view of the liver.

Microarray Analysis. The analysis was performed in the published microarrays obtained from ONCOMINE http://www.oncomine.org in a cohort of 225 HCC human samples versus 200 controls.

Predictive Modeling of HCC outcome Using Nedd8, LKB1 and Akt Signature. Logistic regression was performed to quantify the predictability of Nedd8, LKB1 and Akt model. In the absence of an independent set, we evaluated the performance of the model using leave-one-out cross-validation. ROC-related computation was performed using DiagnosisMed (http://CRAN.R-project. org/package_DiagnosisMed) and pROC package. All computations

were performed using R software (http://expasy.org/tools/ pROC).

Measurements of oxygen consumption rate (OCR) and extracellular acidification rate (ECAR). Primary *Phb1*-KO mouse hepatocytes and BCLC5 cells were seeded respectively in a collagen I coated XF24 cell culture microplate (Seahorse Bioscience), at 2.0 × 10^4 cells per well. After 3 hours, 100 µl of growth media with DMSO or MLN4924 and control or Nedd8 siRNA were added. 48 hours after, growth medium was removed and replaced with 500 µl of assay medium prewarmed to 37°C, composed of DMEM without bicarbonate containing 1 mM sodium pyruvate, 2 mM l-glutamine, and cultured at 37°C in room air. Measurements of oxygen consumption rate (OCR) and extracellular acidification rate (ECAR) were performed after equilibration in assay medium for 1h. After an OCR and ECAR baseline measurement, sequential injections through ports in the XF Assay cartridges of pharmacologic inhibitors: Oligomycin (1mM), an inhibitor of ATP synthase, which allows a measurement of ATP-coupled oxygen consumption through oxidative phosphorylation (OXPHOS); carbonyl cyanide 4-trifluoromethoxy-phenylhydrazone (FCCP) (300 nM), an uncoupling agent that allows maximum electron transport, and therefore a measurement of maximum OXPHOS respiration capacity; and finally Rotenone (1 µM), a mitochondrial complex 1 inhibitor were performed and changes in OCR and ECAR were analyzed. The normalize data were expressed as pmol of O_2 per minute or milli-pH units (mpH) per minute, per µg protein for primary hepatocytes and viability measured by MTT assay for BCLC5 cells.

Liver Metabolomics analysis. Lipid profiles were analyzed as previously described (1, 2). Briefly, four UPLC®/time-of-flight mass spectrometry (TOF)-MS based platforms analyzing methanol; methanol/water and chloroform/methanol liver extracts were combined. Identified ion features in the methanol extract platform included fatty acyls, bile acids, and lysoglycerophospholipids. The extracts prepared for methanol platform were also derivatized for amino acid analysis. The chloroform/methanol extract platform provided coverage over glycerolipids, cholesteryl esters, sphingolipids and glycerophospholipids. Finally, the methanol/water extract platform comprised the study of polar metabolites, such as vitamins, nucleosides, nucleotides, carboxylic acids, coenzyme A derivatives, carbohydrate precursors/derivatives and redox-electron-carriers. For this platform, a mixture of methanol/water (60:40, v/v) containing non-endogenous internal standards was added to liver tissue (50:1, v/w) and homogenized using a Precellys 24 grinder. After 1 hour of incubation at -20ºC samples were centrifuged at 16,000 x g for 15 minutes. The supernatant was collected and chloroform was added. Polar phase was then transferred to a clean tube for solvent evaporation. Dried extracts were resuspended in water and, after centrifugation; supernatants were transferred to vials for UPLC®-MS analysis. Lipid nomenclature follows the LIPID MAPS convention, www.lipidmaps.org.

Metabolomics Data processing and Normalization. Data obtained with the UPLC®-MS were processed with the TargetLynx application manager for MassLynx (Waters Corp.) as detailed (1, 2). Intra- and interbatch normalization followed the procedure described in (3). All the calculations were performed with R v2.13.0 (R Development Core Team, 2010).

Statistical Analysis. Liver metabolite concentrations were compared using unpaired Student´s or Welch´s t test where unequal variances were found. A number of 5 animals per group were studied.

REFERENCES

1. Barr J, Caballería J, Martínez-Arranz I, Domínguez-Díez A, et al . Obesity-dependent metabolic signatures associated with nonalcoholic fatty liver progression. J Proteome Res. 2012;11;2521-2532
2. Martínez-Uña M, Varela-Rey M, Cano A, Fernández-Ares L et al. Excess S-adenosylmethionine reroutes phosphatidylethanolamine towards phosphatidylcholine and triglyceride synthesis., Hepatology. 2013 Mar 6. doi: 10.1002/hep.26399.
3. Van der Kloet FM, Bobeldijk I, Verheij ER and Jellema RH. Analytical error reduction using single point calibration for acute and precise metabolomic phenotyping. J Proteome Res 2009;8:5132-41.

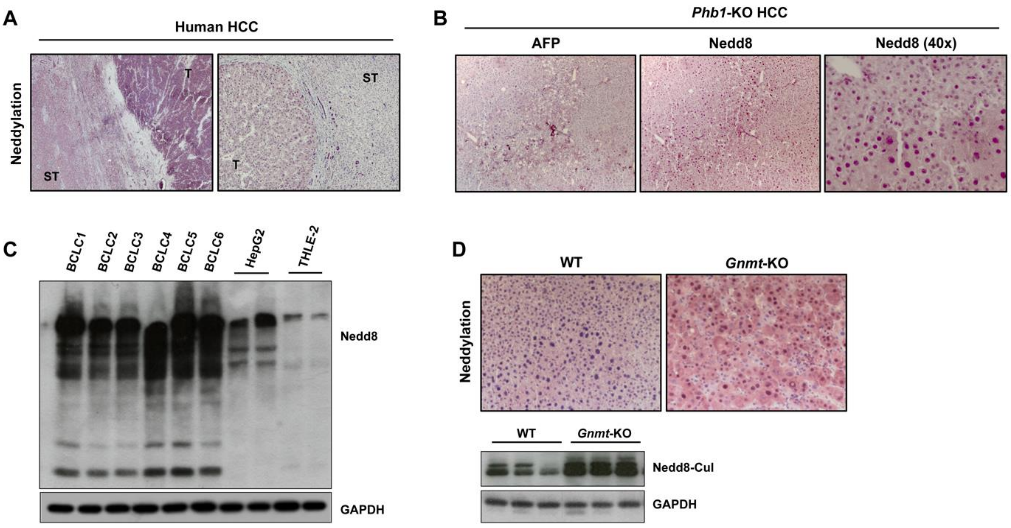

Supplementary Figure 1: Neddylation levels in HCC. (A) Nedd8 IHC analysis in HCC human samples showing its enrichment in tumor areas (T) compared to surrounding tissues (ST). (B) AFP and Nedd8 IHC in *Phb1*-KO liver sections showing an HCC tumor. (C) Western blot analysis of neddylation in different human hepatoma cell lines compared to non-tumoral hepatocytes. (D) Neddylation levels in WT and *Gnmt*-KO livers by IHC and Western blot. Original magnification 10x.

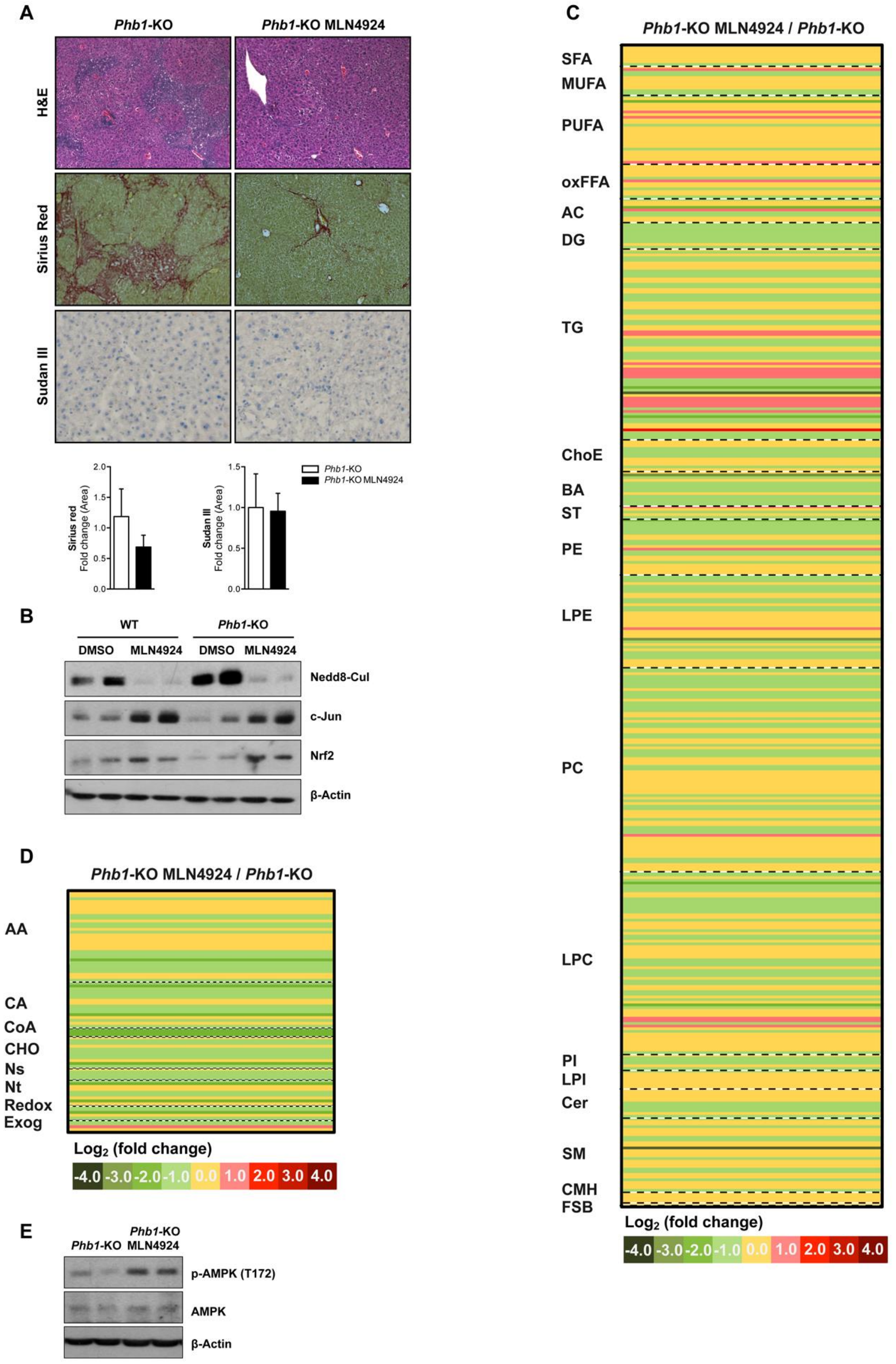

Supplementary Figure 2: MLN4924 treatment effects in *Phb1*-KO mice. (A) H&E, Sirius red and Sudan III staining on liver sections from *Phb1*-KO and *Phb1*-KO treated with MLN4924. Graphical representation of the quantitative analysis for each staining are shown beside. (B) Representative Western blot analysis of neddylation (shown as neddylated cullins), Nrf2 and c-Jun in whole extracts from WT and *Phb1*-KO hepatocytes after 48 hours of MLN4924 treatment. (C) Normalized ion abundance ratios of 510 lipids in liver samples

comparing MLN9424 treated and non-treated *Phb1*-KO mice are shown. For each comparison, log transformed ion abundance ratios are depicted, as represented by the scale. It is relevant to underline that lipids present in this picture have been ordered according to their carbon number and unsaturation degree. Individual metabolite details, including fold-changes and statistical significances, are shown in Supplementary Table VI. (D) Normalized ion abundance ratios of 80 metabolites in liver samples comparing MLN9424 treated and non-treated *Phb1*-KO mice. For each comparison, log transformed ion abundance ratios are depicted, as represented by the color scale. Individual metabolite details, including fold-changes and statistical significances, are shown in Supplementary Table VII. (E) Representative Western blot analysis of AMPK and p-AMPK (T172) in whole liver extracts from *Phb1*-KO mice and *Phb1*-KO mice treated with MLN4924.

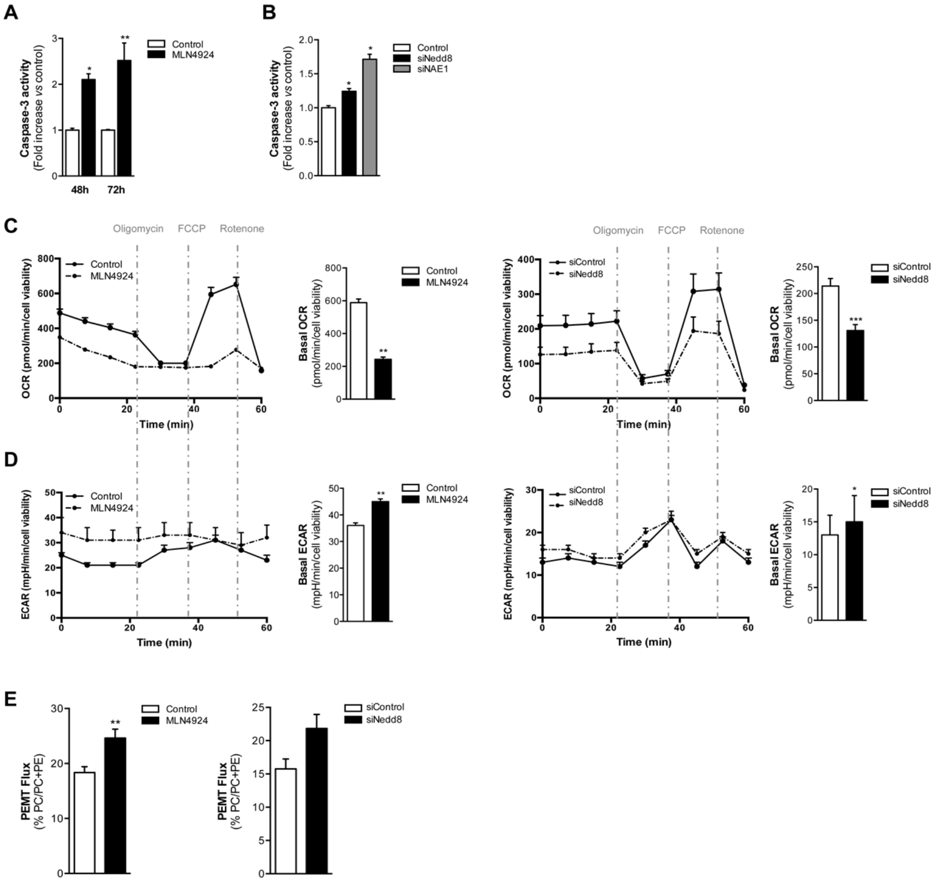

Supplementary Figure 3: Oxidative Phosphorylation and Glycolysis are dependent on neddylation activity in human hepatoma cells. Caspase-3 activity assay of BCLC5 cells (A) treated with MLN4924 for 48 and 72 hours and (B) after Nedd8 and NAE1 silencing. (C) OCR and (D) ECAR were measured in BCLC5 cells after 48 hours of MLN4924 treatment and Nedd8 silencing. The energetic response in cells was measured in the presence of

oligomycin, FCCP and rotenone. (E) PEMT flux in BCLC3 cells after 48 hours of MLN4924 treatment and Nedd8 silencing. The radioactivity incorporated into PC was expressed as a percentage of the radiolabel incorporated into PC plus PE. (Values are mean ± SEM. n=4 samples/time point. *p<0.05; **p<0.01; ***p<0.001 [MLN4924, siNedd8 or siNAE1 *vs* control].

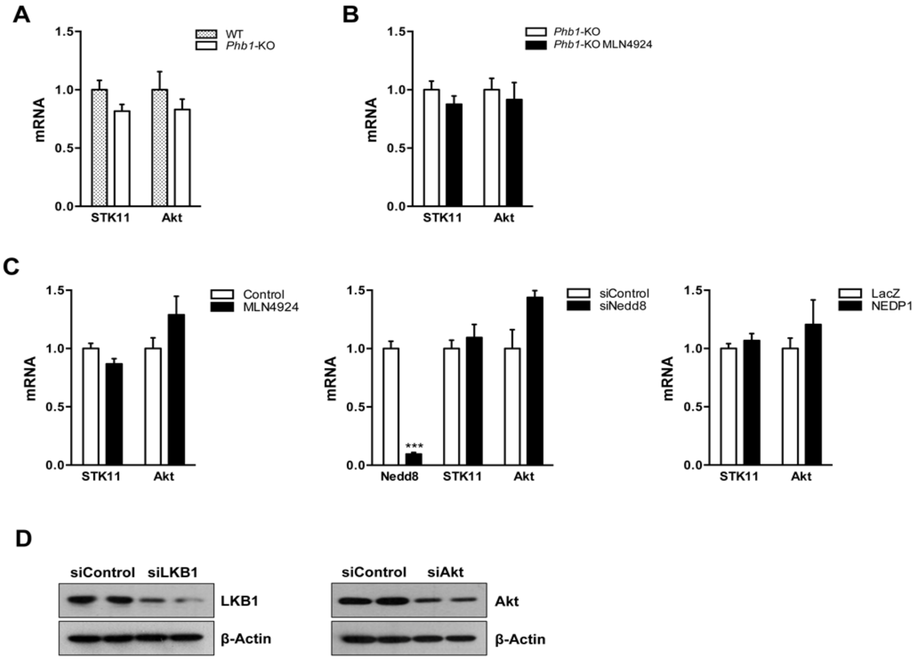

Supplementary Figure 4: LKB1 and Akt transcriptional regulation by neddylation. LKB1 and Akt mRNA expression levels measured by qPCR in (A) WT and *Phb1*-KO livers and (B) *Phb1*-KO and *Phb1*-KO treated with MLN4924 livers. (C) LKB1 and Akt mRNA expression levels measured by qPCR in *Phb1*-KO hepatocytes after MLN4924 48 hours treatment, Nedd8 silencing and NEDP1 overexpression. (D) Representative Western blot analysis showing the silencing of LKB1 and Akt in *Phb1*-KO hepatocytes. ***p<0.001 [siNedd8 *vs* control].

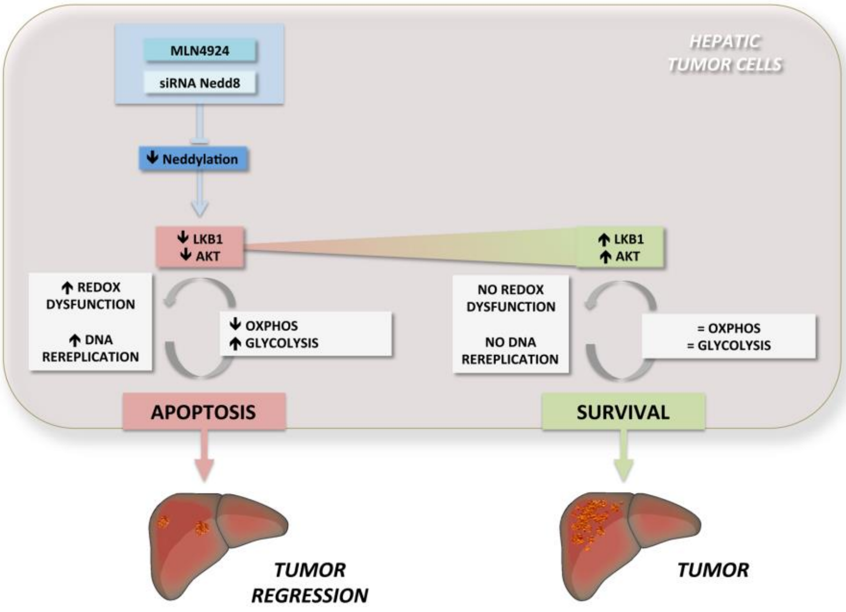

Supplementary Figure 5: Schematic drawing summarizing the role of LKB1 and Akt stabilization on neddylation-induced metabolic disruptions in liver cancer. A. Hepatocytes isolated from Prohibitin 1 (*Phb1*)-KO mice display tumoral characteristics. Neddylation inhibition using the small molecule inhibitor MLN4924 or Nedd8 siRNA accounts for decreased Nedd8 levels in these hepatic tumoral cells. The reduction in Nedd8 levels lowers liver kinase B1 (LKB1) and Akt levels, usually upregulated in tumoral cells. As a consequence of lowering LKB1 and Akt levels, a reduction in the OXPHOS/glycolysis metabolic ratio is observed. Under these circumstances, the metabolic slowdown observed after neddylation inhibition is associated with the negative impact on OXPHOS. As a consequence of decreased OXPHOS, tumoral cells need to look for alternative energetic pathways such as glycolysis. Interestingly, the metabolic switch from OXPHOS to glycolytic flux, usually advantageous to tumoral cells, in here is associated with increased tumoral cell apoptosis. We speculate that this is due to the fact that under neddylation inhibition the cellular energetic pool, already compromised by reduced OXPHOS, is being channeled to the high-demanding energetic process of DNA rereplication, previously shown to be induced under neddylation inhibition. Thereby, neddylation inhibition increased redox dysfunction and increased DNA rereplication account for tumoral cell apoptosis corresponding to the tumor regression observed in *Phb1*-KO mice treated *in vivo* with MLN4924; **B.** On the contrary, boosting the levels of both LKB1 and Akt in tumoral cells was sufficient to counteract the apoptotic response mediated by neddylation inhibition. Indeed, LKB1 and Akt-overexpression accounts for increased OXPHOS/glycolysis ratio neutralizing the decrease in OXPHOS and the elevation in glycolysis described under MLN4924-treatment in *Phb1*-KO hepatocytes where LKB1 and Akt were not overexpressed.

Supplementary Table I: Tumor sizes at week 0 and week 6 in *Phb1*-KO and P*hb1*-KO animals treated with MLN4924.

	Tumor size (mm)	
	**Week 0**	**Week 6**
***Phb1*-KO**	2,2 ± 0,8	2,5 ± 0,9
***Phb1*-KO MLN4924**	2,2 ± 0,5	1,3 ± 0,6

Supplementary Table II: Biochemical analyses of WT control mice and *Phb1*-KO mice after the 6 weeks of MLN4924 treatment.

	**WT**	***Phb*1-KO MLN4924**
**Triglycerides**	134,6 ± 7,5	154,4 ± 30,7
**Glucose**	217,3 ± 7,7	149,8 ± 4,1
**Cholesterol**	143,3 ± 5,0	137,7 ± 11,2
**Total protein**	6,0 ± 0,2	5,9 ± 0,1
**Albumin**	4,0 ± 0,2	3,6 ± 0,1

Supplementary Table III: Significant differences between lipid species levels from livers from MLN4924 treated mice and non-treated *Phb1*-KO animals are shown.

Individual notation	Individual composition or probable ID	Fold-change	p-value
DAG(32:1)	DG(16:0+16:1+0:0)+ DG (14:0+18:1+0:0)	0.50	3.14E-04
DAG(32:2)	DG(14:0+18:2+0:0)	0.67	3.79E-02
DAG(34:2)	DG(16:0+18:2+0:0)	0.71	6.58E-03
TAG(54:5)	TG(18:2+18:2+18:1)	2.01	4.51E-02
TAG(54:6)	TG(18:2+18:3+18:1)	3.05	1.22E-02
TAG(54:7)	TG(18:2+18:3+18:2)	3.32	4.95E-03
TAG(56:7)	TG(22:5+18:2+16:0) + TG(20:4+18:2+18:1)	2.35	2.96E-02
TAG(56:8)	TG(20:5+18:2+18:1) + TG(20:4+18:2+18:2)	3.17	9.11E-03
TAG(56:8)	TG(22:6+18:2+16:0)	3.83	1.73E-02
TAG(58:10)	TG(20:5+20:4+18:1) + TG(20:4+20:4+18:2)	3.65	1.79E-04
TAG(58:9)	TG(20:4+20:4+18:1) + TG(22:6+18:1+18:2)	4.02	1.57E-03
PE(16:0/18:1)		0.46	4.23E-02
PE(40:6)	PE(18:1e/22:5) + PE(20:2e/20:4)	2.97	1.01E-02
PE(16:1)	PE(0:0/16:1)	0.54	1.92E-02
PE(0:0/22:6)		1.31	3.37E-02
PE(20:1e/0:0)		2.10	3.90E-02
PC(32:1)	PC(16:0/16:1)+PC(14:1/18:0)+PC(14:0/18:1)	0.51	1.27E-02
PC(33:1)	PC(15:0/18:1) + PC(16:0/17:1)	0.55	2.10E-02
PC(16:0/18:1)		0.65	2.47E-03
PC(16:0/19:1)		0.67	3.09E-02
PC(17:0/20:4)		1.25	1.87E-02
PC(18:0/20:4)		1.69	4.34E-03
PC(18:0/22:6)		1.89	3.14E-02
PC(34:0e)	PC(O-16:0/18:0) + PC(O-18:0/16:0)	1.98	2.43E-02
PC(18:0/0:0)		1.39	1.38E-02
PC(0:0/16:1)		0.70	5.65E-03
PC(0:0/17:1)		0.62	1.55E-02
PC(0:0/20:5)		0.62	1.29E-02
LPC(16:1)		0.33	1.47E-03
PC(18:1e/0:0)		2.13	1.67E-02
PC(18:1e/0:0)		2.54	3.09E-02
PC(22:1e/0:0)		1.89	2.66E-02
PC(24:2e/0:0)		1.89	3.70E-02
PC(O-16:0/0:0)		1.81	2.59E-02
PC(O-18:0/0:0)		1.81	4.83E-02
LPI(18:0)		1.42	4.15E-02
SM(d18:0/22:0)		0.09	4.00E-02
SM(38:1)	SM(d18:1/20:0) + SM(16:1/22:0)	1.48	5.52E-03
Ratio SM/PC		0.58	4.93E-02
Ratio PC(22:6n-3) to total PC		1.19	1.57E-02

Ratio PC/PE (18:0/20:4)		1.42	3.62E-02
Ratio PC/PE (16:0/18:1)		1.37	1.60E-02

"Individual notation" refers to the confirmed identification of the metabolites. Overlapping of two o more metabolites or non-confirmed identification is indicated in "Individual composition or probable ID". Fold-changes and unpaired Student's t test p-values (or Welch´s t test) were calculated for each comparison considering 5 animals per group. Regarding sphingolipids, dA:B represents the sphingoid base d18:1, sphingosine; d18:2, sphingadiene; d18:0, sphinganine. For glycerophospholipids containing an ether moiety the prefix, O-, denotes the presence of an alkyl ether substituent.

Supplementary Table IV: Central Carbon Metabolism analysis of livers from *Phb1*-KO mice treated with MLN4924 *versus Phb1*-KO.

Fold-changes between metabolite levels and unpaired Student's t test p-values (or Welch´s t test where unequal variances were found) were calculated for each comparison considering 5 animals per group. "Individual notation" refers to the confirmed identification of the metabolites.

Individual notation	Abbreviated name	Fold-change	Student's t-test (p)
Glycine	Gly	0.79	2.48E-02
Glycolic acid		1.45	2.51E-02
Succinate		0.56	5.91E-03
Malonyl-CoA		0.32	5.77E-02

Supplementary Table V: Specific siRNA used for *in vitro* and *in vivo* silencing assays.

Gene name	Species		Sequence
siControl	Homo sapiens	Sense<br>Antisense	5'-UUCUCCGAACGUGUCACAU-3'<br>5'-AUGUGACACGUUCGGAGAA-3'
siNAE1	Homo sapiens	Sense<br>Antisense	5'-GUAGUGUGUUAAUGAUUGA-3'<br>5'-UCAAUCAUUAACACACUAC-3'
siNedd8	Homo sapiens	Sense<br>Antisense	5'-GCCCAGUAAUGUAUGUCUA-3'<br>5'-UAGACAUACAUUACUGGGCAU-3'
siControl	Mus musculus	Sense<br>Antisense	5'-AAUUCUCCGAACGUGUCACGU-3'<br>5'-ACGUGACACGUUCGGAGAAUU-3'
siNedd8	Mus musculus	Sense<br>Antisense	5'-CAUCUACAGUGGCAAGCAA-3'<br>5'-UUGCUUGCCACUGUAGAUGAG-3'
siLKB1	Mus musculus	Sense<br>Antisense	5´-GGGCGGUCAAGAUCCUCAA-3´<br>5´-UUGAGGAUCUUGACCGCCC-3´
siAkt	Mus musculus	Sense<br>Antisense	5´-GCUACUUCCUCCUCAAGAA-3´<br>5´-UUCUUGAGGAGGAAGUAGC-3´

Supplementary Table VI: Optimal Incubation Conditions, Concentration, Reference and Supplier for Each Specific Antibody Analyzed by Western Blotting.

β-Actin	A5441	Sigma	1/5000	TBS-Tween (0.1%)-milk (5%)
Akt	9272S	Cell Signaling Technology	1/1000	TBS-Tween (0.1%)-BSA(5%)
AMPK	07-350	Upstate	1/1000	TBS-Tween (0.1%)-milk (5%)
p-AMPK (T172)	2531S	Cell Signaling Technology	1/1000	TBS-Tween (0.1%)-milk (5%)
c-Jun	9165S	Cell Signaling Technology	1/1000	TBS-Tween (0.1%)-milk (5%)
Flag	F1804	Sigma Aldrich	1/1000	TBS-Tween (0.1%)-milk (5%)
GAPDH	ab8245	Abcam	1/5000	TBS-Tween (0.1%)-milk (5%)
HA	MMS-101R	Covance	1/1000	TBS-Tween (0.1%)
HuR	SC-5261	Santa Cruz Biotechnology	1/5000	TBS-Tween (0.1%)-milk (5%)
LKB1	SC3-32245	Santa Cruz Biotechnology	1/1000	TBS-Tween (0.1%)-BSA(5%)
Nedd8	Y297	Abcam	1/1000	TBS-Tween (0.1%)-milk (5%)
NRF2	SC-722	Santa Cruz Biotechnology	1/5000	TBS-Tween (0.1%)-milk (5%)
V5	R96025	Invitrogen	1/500	TBS-Tween (0.1%)-milk (5%)

Supplementary Table VII: Sequence of primers used for RT-PCR analysis.

Gene name	Symbol	Species		Sequence
18S ribosomal RNA	18S	Homo sapiens	Forward	5'-CCGATAACGAACGAGACTCTGG-3'
			Reverse	5'-TAGGGTAGGCACACGCTGAGCC-3'
Nedd8 Activating Enzime 1	NAE1	Homo sapiens	Forward	5'-TTGTGGCCAAAGAGGGTCAA-3'
			Reverse	5'-ATGATTACCCACAGCGGCAG-3
Neural precursor cell expressed, developmentally down-regulated 8	Nedd8	Homo sapiens	Forward	5'-CTACAGACAAGGTGGAGCGAA-3'
			Reverse	5'-CTCCTCTCAGAGCCAACACC -3'
Serine-threonine kinase 11, Liver Kinase B1	STK11	Homo sapiens	Forward	5'-CGAGGGCAGCTGATGTCGGT-3'
			Reverse	5'-CCGCCCTGCGGCATAAGGTCT-3'
Protein Kinase B Alpha	Akt	Homo sapiens	Forward	5'-ATCCTGGTCCGTCTTCCTC-3'
			Reverse	5'-CTTCCCTAAGCCCCTGGTGA-3'
Glyceraldehyde-3-phospahate dehydrogenase	GAPDH	Mus musculus	Forward	5'- CGTCCCGTAGACAAAATGG-3'
			Reverse	5'- TTGATGGCAACAATCTCCAC -3'
Neural precursor cell expressed, developmentally down-regulated 8	Nedd8	Mus musculus	Forward	5'-CAGCAGCGGCTCATCTACAG-3'
			Reverse	5'-CAGGGCAAGGAGGTAAACGG-3'
Serine-threonine kinase 11, Liver Kinase B1	STK11	Mus musculus	Forward	5'- GTCAGCTGGGGTCACACTTT -3'
			Reverse	5'- TGGTGAAGTCTCCTCTCCCA -3'
Protein Kinase B Alpha	Akt	Mus musculus	Forward	5'- AATGTGGGCTCATGGGTCTG -3'
			Reverse	5'- AGAGGGAGAGGGCCAGTTAG -3'
